# Bad Touch in Childhood is associated with unexplained Gastroenteritis

**DOI:** 10.12669/pjms.38.8.6854

**Published:** 2022

**Authors:** Hina Sultan, Ghulam Ishaq

**Affiliations:** 1 Hina Sultan (PhD) Assistant Professor, Department of Psychology, Lahore Leads University, Lahore, Pakistan; 2 Ghulam Ishaq (PhD) Assistant Professor, Department of Psychology, Institute of Southern Punjab, Lahore, Pakistan

A 12 years old girl was referred by the gastroenterology department to psychiatric department two months back. Gastroenterologist reported that child was suffering from recurrent episodes of abdominal cramps, vomiting, and diarrhea from last one year. Doctors conducted all necessary tests like blood tests, ESR, H-Pylori, Stool test etc. Endoscopy was also done but each medical report did not show any sign of abnormality. Child was less talkative, aggressive, restless, and had increased pulse rate. We, psychologists, tried to make the child feel relaxed and comfortable through *Deep Breathing* and *16 Progressive Muscle Relaxation exercises*. Every week, two sessions were arranged, during 4th session, play therapy including toys, blocks, dolls, drawings, House, Tree, Person; HTP test was introduced. In “Thematic Apperception Test, she was given two cards, the first based on image of a crying girl and the other one comprised of a picture of old man. Child wrote two stories emphasizing that “girl is crying because her servant might look at her private body parts”. In second story, girl wrote, “children should not be left alone with servants”. At this crucial point, therapist asked probing questions and showed unconditional positive regard and empathy for the child which helped the child open-up to share her trauma in detail; revealing being victim of bad touch by her home servant, without penetration. This was quite appalling and a sensitive matter specially for parents as they were unaware regarding actual reason behind their child’s sufferings. So, a range of psychotherapies and family therapies were given and child was returned back to her normal life but the story does not end here. We, psychologists, have encountered almost 30 children during our clinical practice (within six months of our clinical practice) who experienced bad touch by their closed relatives, servants, or peers and children usually manifest their problems in the form of diarrhea, vomiting, and cramps which did not improve by medical examination. Moreover, if we talk about scientific evidence, many studies around the globe including Pakistan have found an association of suspected child sexual abuse with gastrointestinal illnesses.^1^,^2^,

Here, another point is that children might be the victim of sexual assault and rape but could not express it moreover, family members are also unaware or may be offender themselves. As such incidents are not reported due to shame and stigmatization. Children suffer in silence, take pressure on nerves and exhibit physical complaints which are considered socially acceptable. This is extremely tragic that children are not safe even in their homes. So, there is an intense need to educate children about how to escape such unusual incidents. It is also essential to educate the public and health care practitioners as child sexual abuse or even bad touch results in ruining the life of millions of children belonging to different cultures and socioeconomic backgrounds.

## Authors’ Contribution:

**HS** Conceived, designed, & preparation of manuscript. **GI** Critical review and final approval for publication.
